# Octominin: A Novel Synthetic Anticandidal Peptide Derived from Defense Protein of *Octopus minor*

**DOI:** 10.3390/md18010056

**Published:** 2020-01-15

**Authors:** Chamilani Nikapitiya, S.H.S. Dananjaya, H.P.S.U. Chandrarathna, Mahanama De Zoysa, Ilson Whang

**Affiliations:** 1 College of Veterinary Medicine, Chungnam National University, Yuseong-gu, Daejeon 34134, Korea; chamilani14@gmail.com (C.N.);; 2 National Marine Biodiversity Institute of Korea (MABIK), 75, Jangsan-ro 101beon-gil, Janghang-eup, Seochun-gun, Chungchungnam-do 33662, Korea

**Keywords:** anticandidal activity, antimicrobial peptides, *Candida albicans*, Octominin, *Octopus minor*

## Abstract

The rapid emergence of multidrug-resistant pathogens makes an urgent need for discovering novel antimicrobial agents as alternatives to conventional antibiotics. Towards this end, we designed and synthesized a synthetic peptide of 23 amino acids (AAs) (^1^GWLIRGAIHAGKAIHGLIHRRRH^23^) from a defense protein 3 cDNA sequence of *Octopus minor*. The sequence of the peptide, which was named Octominin, had characteristic features of known antimicrobial peptides (AMPs) such as a positive charge (+5), high hydrophobic residue ratio (43%), and 1.86 kcal/mol of Boman index. Octominin was predicted to have an alpha-helix secondary structure. The synthesized Octominin was 2625.2 Da with 92.5% purity. The peptide showed a minimum inhibitory concentration (MIC) and minimum fungicidal concentration (MFC) of 50 and 200 μg/mL, respectively, against *Candida albicans*. Field emission scanning electron microscopy observation confirmed that Octominin caused ultrastructural cell wall deformities in *C. albicans*. In addition, propidium iodide penetrated the Octominin-treated *C. albicans* cells, further demonstrating loss of cell membrane integrity that caused cell death at both MIC and MFC. Octominin treatment increased the production of intracellular reactive oxygen species and decreased cell viability in a concentration dependent manner. Cytotoxicity assays revealed no significant influence of Octominin on the viability of human embryonic kidney 293T cell line, with over 95% live cells in the Octominin-treated group observed up to 100 µg/mL. Moreover, we confirmed the antifungal action of Octominin in vivo using a zebrafish experimental infection model. Overall, our results demonstrate the Octominin is a lead compound for further studies, which exerts its effects by inducing cell wall damage, causing loss of cell membrane integrity, and elevating oxidative stress.

## 1. Introduction

*Candida albicans* is one of the most dominant fungal species of the human microbiota and asymptomatically colonizes in the gastrointestinal and genitourinary tract in healthy people [1]. However, *C. albicans* is an opportunistic pathogen that can cause infections under certain pathological and physiological conditions such as in diabetes, pregnancy, under steroidal chemotherapy, and prolonged broad spectrum antibiotic administration, as well as in patients with acquired immunodeficiency syndrome [2]. Multidrug resistance is considered to be the major cause for failures in candidiasis treatment [3], and several pathogenic strains of *C. albicans* show multidrug resistance against currently used antifungal drugs such as fluconazole, itraconazole, nystatin, caspofungin, ketoconazole, flucytosine, and amphotericin B [4,5]. *C. albicans* shows a diversity of resistance mechanisms such as a decrease in drug accumulation or drug affinity of its targets and changes in drug metabolism [6]. Hence, it is important to identify and develop novel and less toxic anticandidal agents with high therapeutic efficacy to effectively control infections with drug-resistant strains of *C. albicans*. 

Antimicrobial peptides (AMPs) are short peptides that typically consist of less than 50 amino acids (AAs) residues and exhibit antimicrobial activity [7]. To date, over 1200 AMPs have been identified or predicted from bacteria, fungi, plants, invertebrates, nonmammalian vertebrates, and mammals [8,9]. The genes encoding AMPs are highly evolutionarily conserved components of the host defense system against pathogenic microorganisms, and can thus be considered as “natural antibiotics” [10]. AMPs can also be described as “host defense peptides” owing to their additional immunomodulatory functions such as antitumor [11], antiendotoxin [12], cytokine and growth factor [6], immunomodulatory [9], and wound healing functions [13]. The antimicrobial activity of AMPs has been reported against a wide range of pathogens, including bacteria [14], yeast [15], fungi [16], viruses [17], and parasites [18]. Based on the secondary structure, AMPs can be classified into four major groups: α helical, β sheet, loop, and extended peptide [19]. A common characteristic of AMPs is their amphipathic nature with net positive charge due to the presence of multiple residues such as arginine (R), histidine (H) and lysine (K) and hydrophobic residues such as alanine (A), leucine (L), isoleucine (I), tyrosine (Y), and tryptophan (W) [20]. The ability of AMPs to kill microbial pathogens primarily depends on their interaction and binding capacity with the cell membrane or cell wall, which vary according to the net positive charge and ratio of hydrophobic AAs. This amphipathicity of AMPs is an important feature for interacting with cell membranes or their mimics to induce antimicrobial functions, and binding of AMPs to the cell membrane generally changes the membrane permeability and causes nonenzymatic disruption [6], resulting in the efflux of intracellular materials to exert rapid bactericidal/fungicidal activity [21]. 

Although various sources are available for isolating natural AMPs with different degrees of functional activities, it is difficult to obtain sufficiently large quantities of the peptides with the purity required for application in research or for therapeutic purposes. Therefore, the production of synthetic AMPs is becoming an increasingly popular strategy, which can lead to the development of novel therapeutic agents with a wide range of bioactivities [22]. Furthermore, intensifying the effort for the identification and development of novel AMPs is essential to help suppress the rapid spread and continued selection of multidrug-resistant pathogens.

Toward this end, the objective of this study was to screen for AMPs in the transcriptome of *Octopus minor*, and synthesize the peptide to investigate its antifungal effect. A novel AMP was screened and modified based on the defense protein 3 cDNA sequence of *Octopus minor*, which was named Octominin. To evaluate its anticandidal function and mode of action, *C. albicans* was used as a pathogenic model organism, and various parameters of Octominin in the presence of *C. albicans* were determined, including the minimum inhibitory concentration (MIC), minimum fungicidal concentration (MFC), and MFC/MIC ratios, along with the influence of the synthesized protein on the fungal cell, including change of cell membrane structure, cell viability, reactive oxygen species (ROS) production, propidium iodide (PI) uptake. Moreover, to establish the potential of Octominin for development in therapeutic application, we assessed its cytotoxicity in the human embryonic kidney HEK293T cell line. Based on the overall results and possible mode of action, we suggest that Octominin is a potential lead molecule with fungicidal activity that can be developed for commercial use. 

## 2. Results

### 2.1. Designing, Synthesis, and Characteristics of Octominin

**Figure 1:**
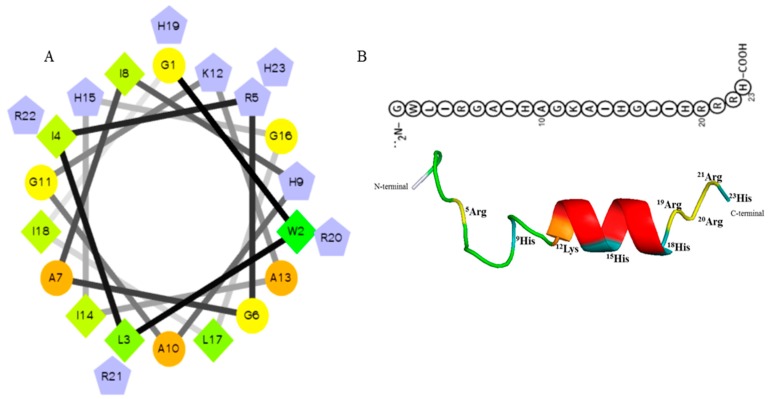
Predicted helical secondary and three-dimensional structures of Octominin. (**A**) Helical wheel of Octominin shows the AAs arrangement and the residue numbers which are counted from the amino (N) terminal of the peptide. The hydrophilic and hydrophobic residues are represented by circles and diamonds, respectively. The potentially charged (positively charged) residues are marked as pentagons in light blue. The most hydrophobic residue is green, and the amount of green is decreasing proportionally to the hydrophobicity, with zero hydrophobicity coded as yellow. (**B**) Three-dimensional structure and AA sequence of Octominin with positively charged residues.

Upon screening of the transcriptome database of *O. minor* for selection of AMPs, defense protein 3 cDNA sequence was considered as one of the candidates. Based on the antimicrobial peptide prediction program [23], and subsequent modification of the sequence fragment, Octominin showed characteristics of known AMPs, including a total net charge of +5, high number of positively charged residues (17% R, 17% H), and hydrophobic residues (17% I, 8% L, 13% A, 4% W) with the total hydrophobic ratio of 43% and protein binding potential (Boman index) of 1.86 kcal/mol. Moreover, the predicted grand average hydropathy value (GRAVY) was −0.2696 and the predicted molecular weight of the peptide was 2626.1 Da. Negatively charged AAs such as aspartate (D) and glutamate (E) were absent in the Octominin sequence, and a total of eight hydrophobic residues were identified on the same surface of the peptide. The predicted secondary and tertiary (three-dimensional) structures of Octominin are shown in Figure 1, including an alpha-helix secondary structure. The molecular weight of synthesized Octominin was 2652.2 Da with 92.5% purity (Supplementary Figure S1). 

### 2.2. MIC, MFC, and Growth Inhibition Profile of C. albicans Exposed to Octominin

**Figure 2:**
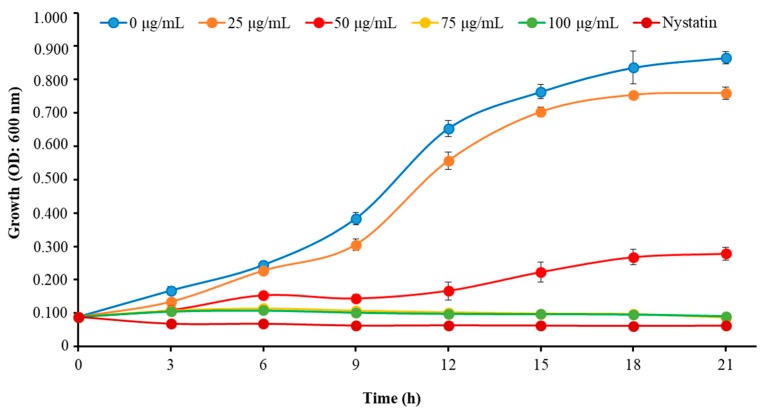
Time–kill kinetics of Octominin against *C. albicans*. *C. albicans* growth was assessed after Octominin treatment (0, 25, 50, 75, and 100 μg/mL) at 3 h intervals by measuring the optical density (OD) at 600 nm. The bars indicate the mean ± standard deviation (*n* = 3).

We determined the MIC and MFC of Octominin to evaluate its ability for inhibiting the growth of *C. albicans* as a candidate anticandidal agent. Octominin demonstrated a clear anticandidal effect with a MIC and MFC of 50 and 200 μg/mL, respectively, representing a MFC/MIC ratio of 4.0. Moreover, the time–kill kinetic analysis revealed clear growth inhibition of *C. albicans* above the MIC level (Figure 2), which was similar to the growth inhibitory effect observed using the known antifungal agent nystatin (10 μg/mL) as a positive control. In addition, Octominin showed partial growth inhibition against *C. albicans* at a lower concentration (25 μg/mL) compared to that of the control. 

### 2.3. Effects of Octominin on Morphological Changes of C. albicans

**Figure 3:**
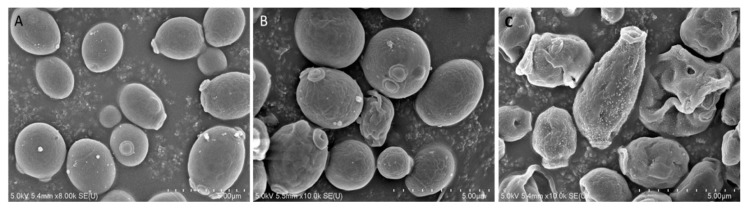
Effect of Octominin on morphological and structural changes of *C. albicans* assessed by field-emission scanning electron microscopy (FE-SEM). (**A**) Untreated *C. albicans*; (**B**) treated with 50 µg/mL (minimum inhibitory concentration; MIC;) Octominin; (**C**) treated with 200 µg/mL (minimum fungicidal concentration; MFC) Octominin.

To investigate the mode of action of Octominin, ultra-structural analysis of *C. albicans* was conducted using field-emission scanning electron microscopy (FE-SEM) after Octominin treatment. The SEM images clearly indicated membrane structural changes of *C. albicans* cells after treatment with Octominin at the MIC and MFC (Figure 3). As expected, untreated *C. albicans* cells displayed a smooth and undamaged cell surface (Figure 3A), whereas, swelling and severe cell wall alterations, including a rough membrane and irregular shape, were observed in the *C. albicans* treated with 50 (Figure 3B) and 200 µg/mL (Figure 3C) Octominin. Moreover, Octominin induced more severe morphological and structural changes at the MFC level than at the MIC level. 

### 2.4. Effects of Octominin on the Viability of C. albicans

**Figure 4:**
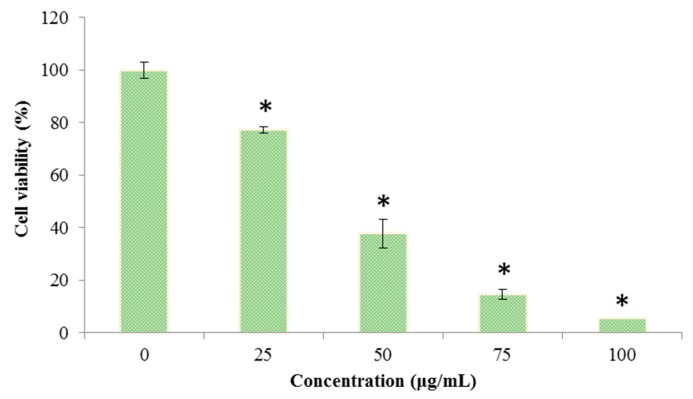
Effect of Octominin on the viability of *C. albicans.* Cell viability was assessed by the 3-(4,5-dimethylthiazol-2-yl)-2,5-diphenyltetrazolium bromide (MTT) assay after treatment with different concentrations of Octominin (0–100 μg/mL) until 24 h. * *P* < 0.05 compared to the control (untreated) group. The error bars indicate the mean ± standard deviation (*n* = 3).

The cell viability of *C. albicans* was determined by a 3-(4,5-dimethylthiazol-2-yl)-2,5-diphenyltetrazolium bromide (MTT) assay after treatment with different concentrations of Octominin (0–100 μg/mL). The cell viability was significantly decreased (*P* < 0.05) when *C. albicans* was treated with Octominin in a concentration dependent manner (Figure 4). The control group showed the highest cell viability, and the lowest cell viability of 9.25% was detected with treatment of Octominin at 100 µg/mL. 

### 2.5. Effect of Octominin on the Membrane Permeability of C. albicans 

**Figure 5:**
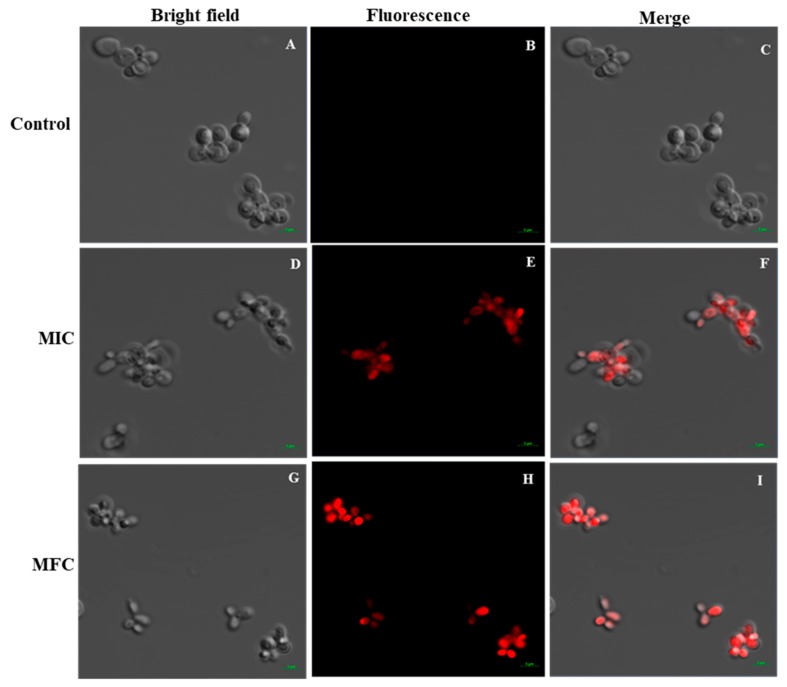
Effect of Octominin on membrane permeability in *C. albicans*. Confocal laser-scanning microscope (CLSM) merged and fluorescence images representing the cell membrane permeability by propidium iodide (PI) staining in *C. albicans* cells treated with Octominin at 30 °C for 6 h. (**A**–**C**) Untreated control; (**D**–**F**) treatment with Octominin at the MIC (50 µg/mL); (**G**–**I**) treatment with Octominin at the MFC (200 µg/mL).

The PI uptake assay was performed to evaluate the effect of Octominin on the membrane permeability of *C. albicans* and its potential association with cell death. PI is an indicator stain that can penetrate dead or damaged cells and subsequently bind with the nucleus, emitting red fluorescence under a confocal laser scanning microscope (CLSM). The results of PI analysis showed that Octominin induced clear membrane damage to the *C. albicans* cells in a concentration-dependent manner (Figure 5). No PI-stained cells were detected in the untreated control group (Figure 5B,C). Both PI-stained and nonstained cells could be observed after treatment of *C. albicans* with the MIC of Octominin (Figure 5E,F), whereas all cells showed the red fluorescence signal after treatment with Octominin at the MFC, indicating the highest degree of membrane permeability (Figure 5H,I). 

### 2.6. Effects of Octominin on ROS Production in C. albicans

**Figure 6:**
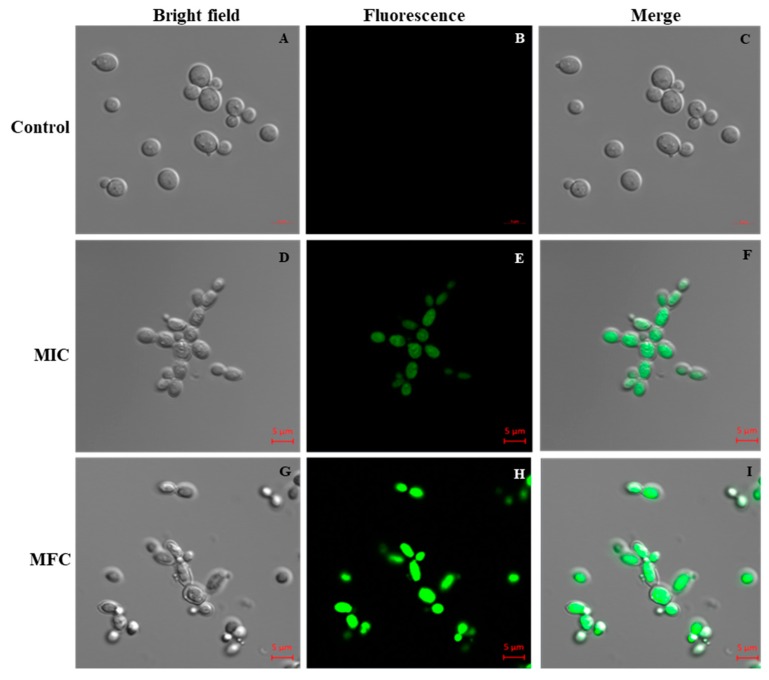
Effect of Octominin on reactive oxygen species (ROS) production of *C. albicans*. CLSM merged and fluorescence images representing ROS production in *C. albicans* with Octominin treatment at the MIC and MFC. (**A**–**C**) Untreated control; (**D**–**F**) treatment with Octominin at the MIC (50 µg/mL); (**G**–**I**) treatment with Octominin at the MFC (200 µg/mL).

To investigate the mechanism of action of Octominin, we examined the change in the level of endogenous ROS production in *C. albicans* after Octominin treatment, which was assessed by staining of the fluorescent probe 5-(and-6)-carboxy-2,7-dihydrodichlorofluorescein diacetate (carboxy-H2DCF-DA). The ROS level was increased in a concentration dependent manner when *C. albicans* was treated with Octominin (Figure 6). Furthermore, *C. albicans* treated with 50 µg/mL (MIC) and 200 µg/mL (MFC) of Octominin showed higher levels of ROS compared to those of untreated cells, indicating that Octominin induced high oxidative stress in the cells. 

### 2.7. Cytotoxicity of Octominin to Mammalian Cells 

**Figure 7:**
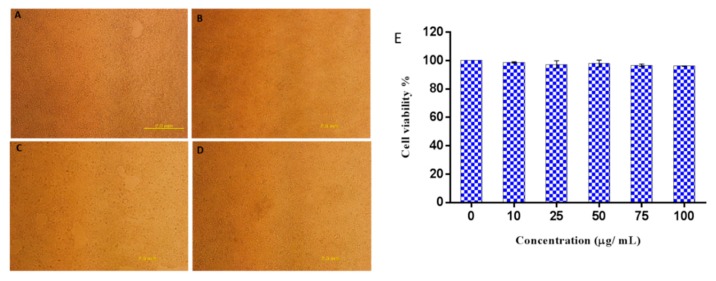
Cytotoxic effect of Octominin on HEK293 cells. (**A**–**D**) Representative images of Octominin-treated HEK293 cells at 48 h post treatment. (**A**) Untreated (control), (**B**) 10 µg/mL Octominin, (**C**) 75 µg/mL Octominin, (**D**) 100 µg/mL of Octominin. (**E**) Cell viability percentage of HEK293 cells treated with Octominin at different concentrations (0, 10, 25, 50, 75, and 100 µg/mL). Data are expressed as the mean ± standard error (*n* = 3).

To further explore the potential safety of Octominin in therapeutic application, its cytotoxicity was tested using human embryonic kidney 293 (HEK293) cells with the EZ-Cytox enhanced cell viability assay kit. No morphological changes of the cells were observed when treated with Octominin up to 100 µg/mL (Figure 7B–D). There was no significant difference in cell viability between the Octominin-treated samples and untreated control (*P* < 0.05), with over 95% of the HEK293 cells remaining viable under all Octominin-treated concentrations up to 100 µg/mL (Figure 7E). Overall, these results confirmed that 100 µg/mL, which inhibits the growth of *C. albicans* by its anticandidal effects, was not toxic to human cells, demonstrating its potential for use in vivo treatments after clinical trials. 

### 2.8. Efficacy of Octominin Treatment on C. albicans Infection in a Zebrafish Model

**Figure 8:**
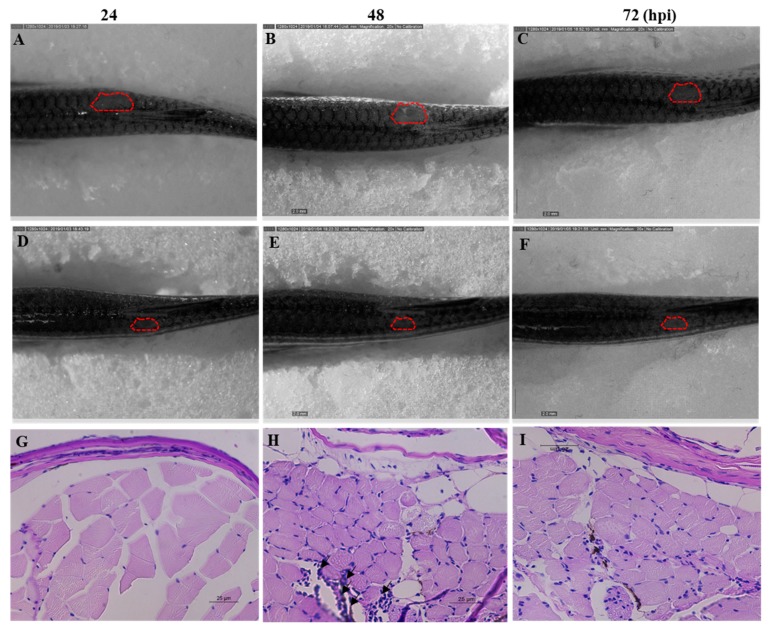
Anticandidal effect of Octominin in *C. albicans*-infected zebrafish. *C. albicans* growth and wound area at 24, 48, and 72 h post treatment of Octominin compared with the control (water-treated) fish. (**A**–**C**) *C. albicans* infected nontreated control. (**D**–**F**) *C. albicans*-infected Octominin treated fish. Effect of Octominin treatment in zebrafish muscle tissue upon *C. albicans* infection evaluated by periodic acid-Schiff-hematoxylin (PAS-H)-stained sections under microscope (400×). Muscle tissues of uninfected (**G**), infected and untreated treated (**H**), and infected and Octominin-treated (**I**) zebrafish. Black arrowhead shows the leukocyte infiltration in the muscle tissue of zebrafish.

*C. albicans* cells were injected to the dorsal muscle of adult zebrafish to study the effectiveness of Octominin treatment under in vivo conditions. The cumulative mortality at 72 h post treatment was 84% and 25% in the control and Octominin-treated (100 µg/fish per day) groups, respectively. Moreover, superficial fungal growth and a wound around the infected site were clearly observed in control (water-treated) fish (Figure 8A–C) at 24, 48, and 72 h post infection (hpi) compared to Octominin-treated fish (Figure 8D–F). In addition, the histopathological data confirmed the treatment effect against *C. albicans*, demonstrating less leukocyte infiltration around the infected site of Octominin-treated fish (Figure 8I) than the infected and untreated fish (Figure 8H) at 72 hpi. 

## 3. Discussion

Discovery of novel AMPs is important to identify potential drug candidates for treating infections with multidrug-resistant pathogens such as *C. albicans*. Several AMPs with diversified antimicrobial functions have been identified from many species of the phylum Mollusk including abalone [14]. We previously reviewed the AMPs in marine mollusks and their potential biomedical applications [24]; thus, the present study represents a further extension of our AMPs research in different mollusk species of the class Cephalopoda. Through screening of the transcriptome database of *O. minor*, we discovered a defense protein 3 cDNA sequence, and a fragment of its AAs sequence was selected and modified, and named Octominin for development of a novel AMP. Octominin shares 92% and 88% identity with two related AMPs namely Chrysophsin-2 and Chrysophsin-1, respectively [25]. We further confirmed that Octominin inhibits the growth of *C. albicans* with a MIC and MFC of 50 and 200 µg/mL, respectively. A chemically synthesized porcine peptide PMAP-23 was reported to have marked antifungal activity (MIC 2–5 µM) against *C. albicans* [26]. In addition, Nikawa et al. [27] synthesized three new cationic peptides with an α-helical structure and amphipathic properties and their abilities to inhibit *C. albicans* were compared to those of known antifungals. The authors suggested that the α-helicity of the peptides has a limited contribution to the killing effect; however, the *p*-value was positively correlated with greater antifungal activity. Although the MIC of Octominin is relatively high, *C. albicans* growth was still delayed by about 3 h (in time–kill kinetics) as same to the 100 µg/mL. Moreover, 50 and 100 µg/mL of Octominin treatment resulted 35% and 5% cell viability at 24 h, respectively. This indicates that the viable cell number decreases rapidly under Octominin treatment, suggesting the efficient killing of the *C. albicans* cells. Therefore, the anticandidal activity of Octominin could be associated with its amphipathic nature, total hydrophobicity (43%), and net positive charge (+5). 

Several peptides have been reported to cause ultrastructural damage to the cell wall of *C. albicans*. For example, a short peptide bovine lactoferrin (FKARRWQWRM) showed efficient inhibitory activity against *Candida* by damaging the cell wall via direct interaction with the cell surface, followed by induction of the autolytic response [28]. Moreover, the chemically synthesized HP (2-20) peptide derived from the *N*-terminus *Helicobacter pylori* ribosomal protein L1 showed free diffusion of PI suggesting that the *C. albicans* cell membrane was damaged [29]. Similarly, PMAP-23 exerts its antifungal activity by creating pores in the cell membrane rather than to the cell wall [26]. Another synthetic hybrid peptide, HP(2-9)-magainin 2(1-12), showed strong antifungal activity via damage to the fungal cell membrane, resulting in K^+^ release from the cells [18]. The FE-SEM images of Octominin-treated cells clearly demonstrated its ability to damage the cell wall and the membrane based on the high number of malformed cells. Moreover, free diffusion of PI into the cytoplasm of *C. albicans* suggested that Octominin might have strong damaging effects on the *C. albicans* cell wall and/or cytoplasmic membrane. 

Furthermore, we demonstrated that at least part of the mode of action of Octominin in *C. albicans* cells was associated with the accumulation of ROS based on oxidation of the fluorescent dye DCFH-DA (an indicator of ROS). The formation of ROS such as superoxide anion (O_2_^−^), hydroxyl radical (^–^OH), hydrogen peroxide (H_2_O_2_), and singlet oxygen (^1^O_2_) can lead to oxidative stress in cells, consequently inducing damage to the macromolecules such as DNA, RNA, and proteins. A state of oxidative stress could activate degradation of the structural components, i.e., cellular membranes, and inactivate their basic functions by increasing the permeability, ultimately resulting in exposure of the cellular contents to the extracellular environment [30]. Indeed, the formation of ROS has been suggested as a critical step in the fungicidal mechanism of several AMPs [31]. However, Veerman et al. [32] suggested that ROS production did not play a role in the killing effect of the human salivary peptide histatin 5 in *C. albicans*. Moreover, histatins bind to a receptor on the fungal cell membrane and enter the cytoplasm, subsequently the mitochondrion, suggesting a role in oxidative stress [33]. Thus, the precise mode of action of some of the antifungal peptides against *C. albicans* remains controversial or unclear. 

Based on its physico-chemical properties (alpha-helical, amphipathic, hydrophobic, and cationic), FE-SEM images, PI uptake, and ROS accumulation, we suggest that Octominin has multifactorial effects on *C. albicans,* resulting in structural disruption of the cell wall and the membrane to ultimately kill the fungal cells. We propose that Octominin may directly interact with the cell wall and then the phospholipid bilayer of the fungal cell membrane, leading to alterations of lipid organization in the membrane or the formation of ion-permeable channels (i.e., pores) in the membranes allowing for diffusion of the cell contents. Further, Octominin might directly interact with the cell membrane of *C. albicans* to disrupt its function and enhance its permeability. Octominin could then reach intracellular targets, the nucleus and DNA, leading to apoptosis. However, the exact mechanism by which Octominin exerts its fungicidal activity will need to be explored and identified in future studies. 

One of the crucial problems associated with the development of an antifungal therapeutic drug is the cytotoxicity of the peptide that limits its safety and application. Here, we conformed that Octominin does not influence the viability of mammalian cells. Further, the overall results of in vivo experiments with a zebrafish infection model suggested that Octominin could effectively reduce the *C. albicans* infection; however, complete recovery was not observed, which may have been due to loss of some Octominin in the water after the treatment. Nevertheless, this result suggests that Octominin has good potential for triggering the host defense against *C. albicans,* supporting its functional novelty. Moreover, Octominin can be used safely as an anticandidal agent against *C.* *albicans* with maximum efficacy. However, its safety and mechanism of action need to be elucidated more detail in future studies. 

In summary, although the properties of Octominin are somewhat inconsistent with those of previously reported anticandidal peptides, the basic characteristics as an AMP were confirmed. The net positive (+5) charge of the novel peptide Octominin leads to its affinity towards the lipid plasma membrane and specificity for *Candida*, and favors the interaction towards the anionic components in the *C. albicans* plasma membrane. Octominin showed a potent anticandidal activity without mammalian cell toxicity, demonstrating its potential for development as a novel anticandidal therapeutic agent. Despite expanding knowledge of antifungal peptides in recent years, this study demonstrates the untapped potential for discovering novel effective antifungal compounds, which can help to overcome current challenges of the treatment of drug resistant infections.

## 4. Materials and Methods 

### 4.1. Design and Synthesis of the Screened AMP in O. minor

We screened the immune functional gene sequences from our *O. minor* transcriptome database for selection of candidate AMPs. The defense protein 3 cDNA sequence was selected and submitted to the National Center for Biotechnology Information (NCBI) (https://www.ncbi.nlm.nih.gov/) database under the accession number MN876862. The *N*-terminal region of the defense protein 3 AA sequence was selected as a template for designing the Octominin peptide using the prediction tool of the antimicrobial peptide database (APD) (http://aps.unmc.edu/AP), [23] according to the criteria of hydrophobicity, net charge, total hydrophobic ratio, and protein binding potential. We modified the selected sequence by increasing the number of hydrophobic (2 to 4 I, 1 to 2 L, 1 to 3 A, and 0 to 1 W) and positively charged residues (3 to 4 R and 0 to 4 H) to obtain the characteristic features of an AMP, resulting in the novel 23-AA Octominin peptide (^1^GWLIRGAIHAGKAIHGLIHRRRH^23^). The peptide was synthesized by a solid-phase peptide synthesis technique (AnyGen Co., Gwangju, Korea) and purified by reverse phase high performance liquid chromatography using a SHIMADZU C18 analytical column (Shimadzu HPLC LabSolution, Kyoto, Japan). 

### 4.2. Culture of C. albicans 

We used the *C. albicans* strain KCTC 27242 from the Korean Collection for Type Culture. A single colony picked up from a potato dextrose agar (PDA) plate was cultured in potato dextrose broth (PDB) under aerobic conditions at 30 °C for 24 h in a shaking incubator at 180 rpm. The *C. albicans* culture was centrifuged at 3500 rpm for 10 min to harvest the cells and washed twice with phosphate buffered saline (PBS, pH 7.4) followed by re-suspension in PBS (OD_600_ 0.1) to adjust the concentration of 10^6^ colony forming units per milliliter (CFU/mL), which was measured using a hemocytometer [30]. 

### 4.3. Determination of MIC and MFC of Octominin for C. albicans 

The MIC and MFC levels of Octominin on *C. albicans* were determined using the broth microdilution susceptibility test and subculture, respectively according to the guidelines of the Clinical and Laboratory Standards Institute (CLSI, 2008 M27-A3). Different concentrations (0–200 μg/mL) of Octominin were tested with 10 μg/mL nystatin (Sigma-Aldrich, Saint Louis, USA) as a standard antifungal drug (positive control) [33]. The MIC was determined as the minimal concentration required to inhibit the visual growth of *C. albicans*. The MFC was determined by sub-culturing 10 μL of the medium collected from the wells showing no microscopic growth after 48 h on PDA medium plates, representing the lowest concentration that yielded no colonies after 24 h growth on agar. The calculated MFC/MIC ratio was used to determine whether the AMPs Octominin had more fungistatic (MFC/MIC ≥ 4) or fungicidal (MFC/MIC < 4) activity according to previously reported criteria [34]. For the time kill kinetic analysis, *C. albicans* (10^6^ CFU/mL) was incubated with Octominin at various concentrations (0–100 μg/mL) in PDB at 30 °C, and the inhibitory effects were investigated after incubation at different time intervals (0, 3, 6, 9, 12, 15, 18, and 21 h) by measuring the absorbance at 600 nm using a microplate reader (Bio-Rad, Saint Louis, USA). The time–kill analysis experiment was performed in triplicate.

### 4.4. Determination of the Effect of Octominin on C. albicans Cell Viability

The effects of Octominin on the viability of *C. albicans* were tested by MTT assay as described by Dananjaya et al. [30]. In brief, *C. albicans* was cultured in PDB (1 × 10^6^ CFU/mL) and treated with the different concentrations of Octominin at 30 °C for 24 h in a shaking incubator at 180 rpm. The culture was then centrifuged at 3500 rpm for 10 min and the cells were washed with PBS. For the cell viability assay, *C. albicans* cells were reacted with 20 μL of MTT reagent (Sigma Aldrich, Saint Louis, USA) for 30 min, and the samples were re-suspended with dimethyl sulfoxide (Sigma Aldrich, Saint Louis City, USA). The cell viability was calculated based on the OD at 570 nm using a microplate reader (Bio- Rad, Saint Louis, USA).

### 4.5. Analysis of Morphological Changes of C. albicans upon Octominin Treatment

To investigate the effect of Octominin on the cell morphology of *C. albicans*, FE-SEM analysis was conducted as described by Dananjaya et al. [30] with some modifications. *C. albicans* cells (10^6^ CFU/mL) were treated with Octominin at the MIC (50 μg/mL) and MFC (200 μg/mL) for 6 h. As a control, *C. albicans* cells were left untreated and cultured for the same period. The cells were pelleted, washed with PBS, and prefixed with 2.5% glutaraldehyde for 30 min. The prefixed cells were then washed with PBS, and serially (30%, 50%, 70%, 80%, 90%, and 100%) dehydrated using ethanol. The fixed cells were dried and coated with platinum using an ion sputter (E-1030, Hitachi, Japan). Treated and control *C. albicans* cells were observed by FE-SEM (Sirion FEI, Eindhoven, The Netherlands).

### 4.6. Determination of the Effect of Octominin on the Membrane Permeability of C. albicans 

To determine the effect of Octominin on membrane permeability, a PI uptake assay was conducted as described by Dananjaya et al. [30]. PI is a red-fluorescent nuclear and chromosome counterstain that indicates changes of cell membrane permeability, and is commonly used to detect the presence of dead cells. Octominin-treated (MIC; 50 μg/mL and MFC; 200 μg/mL levels) and control cell suspensions of *C. albicans* were centrifuged (5000 rpm for 2 min) and the pellets were resuspended in PBS. The cells were then incubated with 5 μg/mL of PI (Sigma Aldrich, Saint Louis, USA) at 30 °C for 15 min in the dark, and then the excess stain was washed with PBS. Finally, one drop of each suspension was placed on a cover slip, and fluorescence images were observed using a CLSM with a scan head integrated to the Axiovert 200 M inverted microscope (Carl Zeiss, Jena, Germany). The PI fluorescence emission was recorded at 585 nm. 

### 4.7. Effect of Octominin on ROS Production in C. albicans

Accumulation of ROS in *C. albicans* was quantified using fluorescent probe carboxy-H2DCF-DA (Invitrogen, Carlsbad, USA). In brief, *C. albicans* culture (10^6^ CFU/mL) was treated with Octominin (MIC; 50 μg/mL and MFC; 200 μg/mL) and incubated at 30 °C for 6 h. The cells were then harvested by centrifugation at 5000 rpm for 2 min. To detect ROS levels, the cells were stained with H_2_DCF-DA (30 μg/mL) and incubated for 30 min at room temperature (24 ± 1 °C) followed by centrifugation at 5000 rpm for 2 min. The cells were washed with ×1 PBS and the dichloro-fluorescein (DCF) fluorescence was measured using CLSM (LSM5 Live Configuration Variotwo VRGB, Zeiss, Germany) at an excitation wavelength of 488 nm and an emission wavelength of 535 nm. 

### 4.8. Analysis of Octominin Cytotoxicity on Mammalian Cells 

To determine the cytotoxicity of Octominin, HEK293T cells (American Type Culture Collection ATCC-11268) were treated with Octominin, and the cell viability was assessed. In brief, HEK293T cells were seeded in Dulbecco’s modified Eagle’s medium (Invitrogen, Carlsbad, USA) with 1% antibiotic/antimycotic solution (Glibco, Carlsbad, USA) and 10% fetal bovine serum (Hyclone, Carlsbad, USA). The cells were then seeded in 96-well flat bottom microtiter plates at a density of 1.5 × 10^4^ cells per well with 100 μL of medium, and incubated at 37 °C in a 5% CO_2_ atmosphere. After 24 h of culture, the medium was aspirated out, and the cells were washed with PBS. Each well was treated with different Octominin concentrations (0–100 µg/mL). Untreated cells were used as a control. Ultimately, cell viability was determined at 48 h post treatment using EZ-Cytox Enhanced cell viability assay kit (DoGenBio Co., Seoul, Korea) following the manufacturer’s protocol.

### 4.9. In Vivo Efficacy of Octominin upon C. albicans Infection in a Zebrafish Model

#### 4.9.1. Zebrafish Husbandry 

All zebrafish experiments were conducted in accordance with the institutional animal ethics guidelines and under supervision and approval of the committees of Chungnam National University (CNU-00866). Wild-type AB zebrafish were maintained under standard culturing conditions using an automated water recirculating system (14 h:10 h light:dark cycle at 28 ± 0.5 °C, conductivity of 500 + 50 µS/sm, feeding equivalent to 4% of their body weight per day) throughout the experimental period. 

#### 4.9.2. Determination of In Vivo Efficacy of Octominin upon *C. albicans* Infection 

The effectiveness of Octominin as an antifungal agent was tested using a zebrafish model of *C. albicans* infection according to the method described by Kulatunga et al. [35]. In brief, a total of 18 zebrafish (average weight: 0.35 ± 0.05 g) were divided into three groups: (1) Uninfected control with water treatment (Control), (2) *C. albicans* infected and water treated (Water treated), and (3) *C. albicans*-infected and Octominin-treated (Octominin-treated). The fish were anesthetized using system water containing 160 μg/mL of buffered tricaine (Ethyl 3-aminobenzoate methanesulfonate). *C. albicans* (4 μL) was injected intramuscularly into the dorsal muscle of the water-treated and Octominin-treated zebrafish groups at a dose of 1 × 10^6^ cells/fish using Hamilton^®^syring (10 μL). Fish in the control group were injected with 4 μL of autoclaved distilled water. Twenty microliters of Octominin (100 μg/fish) was treated to the fish as a topical application by placing it at the site of fungal injection. Treatment was administered after the infection (0 h) and then every 24 hpi up to 72 hpi. After the treatment, fish were kept outside for 3 min to allow for Octominin absorption to the infection site and then released back to the aquarium tanks. The water-treated group received the same treatment but with the same volume of water. All fish were kept in individual tanks during the experiment period. To confirm the antifungal effect of Octominin, photographs of both the water- and Octominin-treated fish were acquired for comparing the infected/healed area at 24, 48, and 72 hpi. For histological examinations, fish were euthanized with an over dose of tricaine, and then the muscle tissues at the infection site were collected and preserved in 10% neutral buffered formalin for 24 h. Formalin fixed muscle tissues were processed (Leica^®^ TP1020 Semi-enclosed Benchtop Tissue Processor, Nussloch, Germany), embedded (Leica EG1150 Tissue Embedding Center, Nussloch, Germany), and sectioned into 4 μm thickness (Leica RM2125 microtome, Nussloch, Germany). The tissue samples were stained by standard PAS staining. Briefly, the sections were deparaffinized in xylene, and rehydrated in a series of ethanol using, distilled water. The sections were then treated with periodic acid (0.5 mg/mL), washed well in distilled water, and stained with Schiff’s reagent. Stained sections were then washed well in fast running lukewarm tap water, the nuclei were stained with hematoxylin, and the sections were rinsed in tap water followed by differentiation in acid alcohol and bluing in Scott’s tap water. Finally, the sections were dehydrated in a series of ethanol, cleared in xylene, and mounted with a xylene based medium. Sections were examined under a light microscope (Leica 3000 LED, Wetzlar, Germany) and the images were captured with a Leica DCF450-C camera (400×).

### 4.10. Statistical Analysis

All experimental data were analyzed using GraphPad Prism software version 5 (GraphPad Software Inc., La Jolla, USA). One way of analysis of variance and unpaired two-tailed t-test were performed to find the significant (*P* < 0.05) differences between controls and peptide treated samples and the data are presented as means ± standard deviations for replicates. 

## Supplementary Materials

The following are available online at https://www.mdpi.com/1660-3397/18/1/56/s1, Supplementary Figure S1.
