# Supplementary material for: Octominin: A Novel Synthetic Anticandidal Peptide Derived from Defense Protein of Octopus minor
Source: Mar Drugs. 2020 Jan 15;18(1):56. doi: 10.3390/md18010056 (PMC7024321; doi:10.3390/md18010056)
Supplement: Supplementary file 1 [file marinedrugs-18-00056-s001.pdf]

Supplementary

Octominin: A Novel Synthetic Anticandidal Peptide Derived from Defense Protein of *Octopus minor*

Chamilani Nikapitiya[1], S.H.S. Dananjaya[1], H.P.S.U. Chandrarathna[1], Mahanama De Zoysa[1,*] and Ilson Whang[2,*]

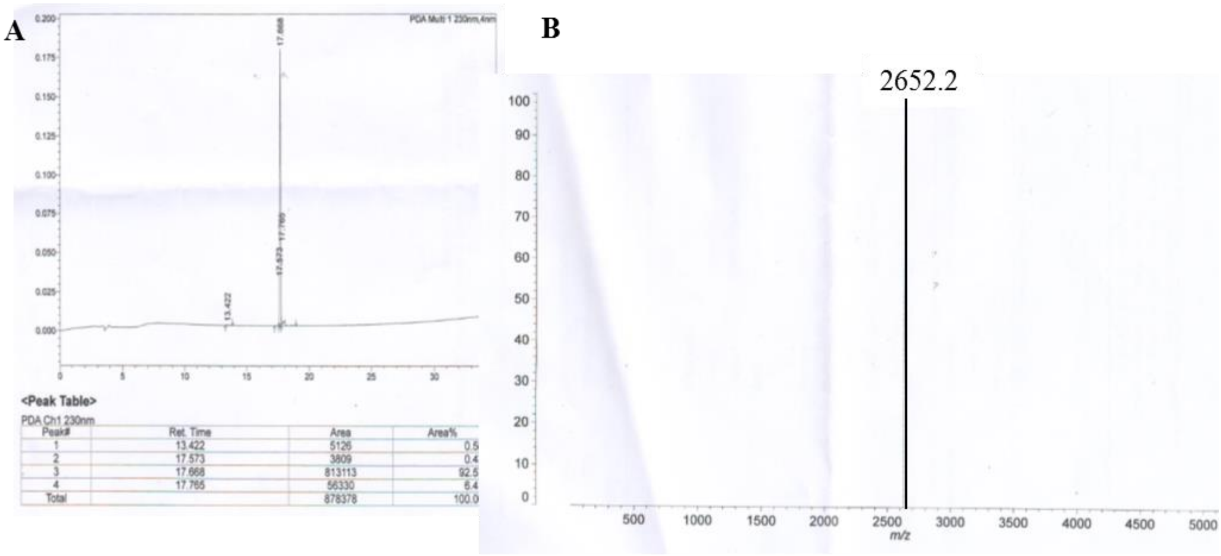

Supplementary figure 1: Purity and molecular weight of synthesized Octominin.

A) Chromatogram of synthesized Octominin showing the level of purity; B) molecular weight (Da).
